# Tracing the Religious Life Course: Intergenerational Sources of Later Life Religiosity

**DOI:** 10.1093/geroni/igaa057.2268

**Published:** 2020-12-16

**Authors:** Merril Silverstein, Woosang Hwang, Joseph Blankholm

**Affiliations:** 1 Syracuse University, Syracuse, New York, United States; 2 University of California, Santa Barbara, Santa Barbara, California, United States

## Abstract

The development of religiosity in later life has its origins in earlier phases of the life course, yet few studies have investigated the contribution of early forms of religious exposure to religious beliefs and behaviors in old age. This investigation uses multigenerational data from the Longitudinal Study of Generations taken from 385 baby-boom children age 16-26 and their parents, linked to religious orientations of these children in midlife and old age. Relying on the “chains of risk” perspective, we found that parental religious intensity in 1971 strengthened their children’s behavioral and cognitive religiosity in later life through their indirect effects on children’s early and midlife religiosity. Our results demonstrate both intergenerational and life course forms of stability in religious belief and practice. Evidence suggests that parental influence creates religious momentum in their children that carries from adolescence/young adulthood through the unfolding of human lives into old age. Part of a symposium sponsored by the Religion, Spirituality and Aging Interest Group.

